# Heterotopic pancreas in the gastrointestinal tract in children: a single-center experience and a review of the literature

**DOI:** 10.1186/s13052-019-0738-3

**Published:** 2019-11-09

**Authors:** Giorgio Persano, Noemi Cantone, Elisa Pani, Enrico Ciardini, Bruno Noccioli

**Affiliations:** 1 Department of Pediatric Surgery, IRCCS Gaslini, via Gerolamo Gaslini, 5, 16147 Genoa, Italy; 2 Department of Neonatal and Emergency Surgery, Meyer Children’s Hospital, Florence, Italy; 3 School of Pediatric Surgery, University of Genoa, Italy - Department of Pediatric Surgery, Meyer Children’s Hospital, Florence, Italy; 4 Department of Pediatric Surgery, District Hospital, Trento, Italy

**Keywords:** Heterotopic pancreas, Ectopic pancreas, Symptomatic heterotopic pancreas

## Abstract

**Background:**

Heterotopic pancreas, that is the abnormal localization of a well-differentiated pancreatic tissue, is a rare occurrence in pediatric patients. Most lesions are found incidentally; in some circumstances, the presence of heterotopic pancreas may cause gastrointestinal symptoms, such as obstructive symptoms or bleeding.

**Patients and methods:**

The clinical notes of patients with histological diagnosis of heterotopic pancreas treated at Meyer Children’s Hospital between 2009 and 2017 have been retrospectively examined.

Four variables have been examined: clinical presentation, age at diagnosis, timing of surgery and localization of the heterotopic pancreas. Patients have been classified accordingly.

**Results:**

Fourteen patients were diagnosed with heterotopic pancreas at a single institution. In half cases, heterotopic pancreas caused symptoms that warranted surgical exploration. Symptomatic patients were significantly older than patients in whom heterotopic pancreas was an incidental finding (mean age 9 years and 5 months vs 2 years and 9 months; *p* = 0.02). Heterotopic pancreas was more frequently found in patients who underwent urgent surgical procedure than in patients who underwent elective surgery (2.61% vs 0.22%; *p* < 0.0001). In all cases, foci of heterotopic pancreas were resected.

**Conclusion:**

Heterotopic pancreas is usually discovered in the submucosa of the stomach, duodenum and small bowel. Heterotopic tissue may cause symptoms related to mechanical complications, bleeding from the surrounding intestinal mucosa or, occasionally, to the development of malignancy.

Heterotopic tissue is a rare but clinically relevant cause of gastrointestinal symptoms. The presence of heterotopic tissue should be considered in children with gastrointestinal symptoms of unclear origin and surgical resection is advisable.

## Background

The term “heterotopic pancreas” refers to the abnormal localization of a well-differentiated pancreatic tissue, without anatomical and vascular connection with the normal pancreas [1]. In the general population, the incidence heterotopic pancreas has been reported between 0.55 and 13.7% in autopsy series [2, 3]and between 0.18% [4] and 5.3% in the most recent surgical series [5].

Heterotopic pancreas is usually located in the upper gastrointestinal tract, the commonest sites being the stomach and the duodenum, followed by Meckel’s diverticulum and, less commonly, the jejunum and the ileum; other sites are rarely reported [4].

In large series, heterotopic pancreas is asymptomatic in approximately 85% of patients and lesions are diagnosed incidentally during procedures motivated by other indications [4, 5]; however, other authors report foci of heterotopic pancreas causing symptoms that warrant surgical exploration in over 35% of patients [6, 7]. In pediatric series, some authors report symptomatic heterotopic pancreas in only 8% of cases [8], while others report symptoms in over 60% patients who are found to have heterotopic pancreas [9].

Considering this high variability in the available data, optimal management has not been established.

Here we report the experience of Meyer Children’s Hospital with heterotopic pancreas between 2009 and 2017 and a review of the literature.

## Patients and methods

We retrospectively examined the clinical notes of patients with histological diagnosis of heterotopic pancreas that have been treated at Meyer Children’s Hospital between 2009 and 2017.

Four clinical variables have been examined: clinical presentation, age at diagnosis, timing of surgery and localization of the heterotopic pancreas. Patients have been classified accordingly.

Regarding clinical presentation, patients whose presenting complaints were caused by heterotopic pancreas were classified as “symptomatic”, while patients in whom heterotopic pancreas was found during surgical exploration for other indications were classified as “incidental”. Patients that had symptoms related to mechanical complications of Meckel’s diverticulum, such as intussusception, or related to mechanical complications of bowel duplication that were independent from the presence of heterotopic pancreas were classified as “incidental”.

Regarding age at diagnosis, patients have been classified as younger than 1-year vs older; the 1-year cut-off has been established because the most common indications for surgical exploration significantly vary between population younger than 1 year and older [10, 11], thus justifying the distinction in two groups. Mean age at diagnosis has been calculated separately in the two subgroups of clinical presentation (i.e. “symptomatic” and “incidental”) and data have been compared using Student’s t-test (Prism software; GraphPad Software, Inc., San Diego, CA). A value of *p* < 0.05 was considered statistically significant.

Regarding timing of surgery, patients were divided into two subgroups, “urgent” (i.e. patients undergoing surgical procedure within 24 h from first evaluation) or “elective” (i.e. patients undergoing surgical procedure more than 24 h from first evaluation).

Regarding localization, four subgroups have been identified: “gastro-pyloric” (i.e. patients who had heterotopic pancreas proximal to the pyloric vein), “small bowel” (i.e. patients who had heterotopic pancreas in otherwise normal bowel between the ligament of Treitz and the ileo-cecal valve), “Meckel’s diverticulum” (i.e. patients who had a Meckel’s diverticulum that contained heterotopic pancreas) and “duplication” (i.e. patients who had an intestinal duplication that contained heterotopic pancreas).

Categorical data were compared using Fisher’s test (Prism software; GraphPad Software, Inc., San Diego, CA). A value of *p* < 0.05 was considered statistically significant.

Histology specimens were classified according to the Heinrich classification as modified by Gaspar-Fuentes et al into four types: type I, with all pancreatic representatives (complete heterotopia); type II, composed of ducts only (canalicular heterotopia); type III, with only acini (exocrine heterotopia); and type IV, only with the islets of Langerhans (endocrine heterotopia) [3, 12]. Additional histologic features has also been described.

## Results

In the examined period, 14 patients were diagnosed with heterotopic pancreas. During the same period, 2600 patients underwent formal videolaparoscopic or laparotomic exploration of the whole bowel for various indications, such as obstruction, malrotation, intussusception or gastrointestinal bleeding: the incidence of heterotopic pancreas can be estimated as approximately 0.54%.

**Fig. 1:**
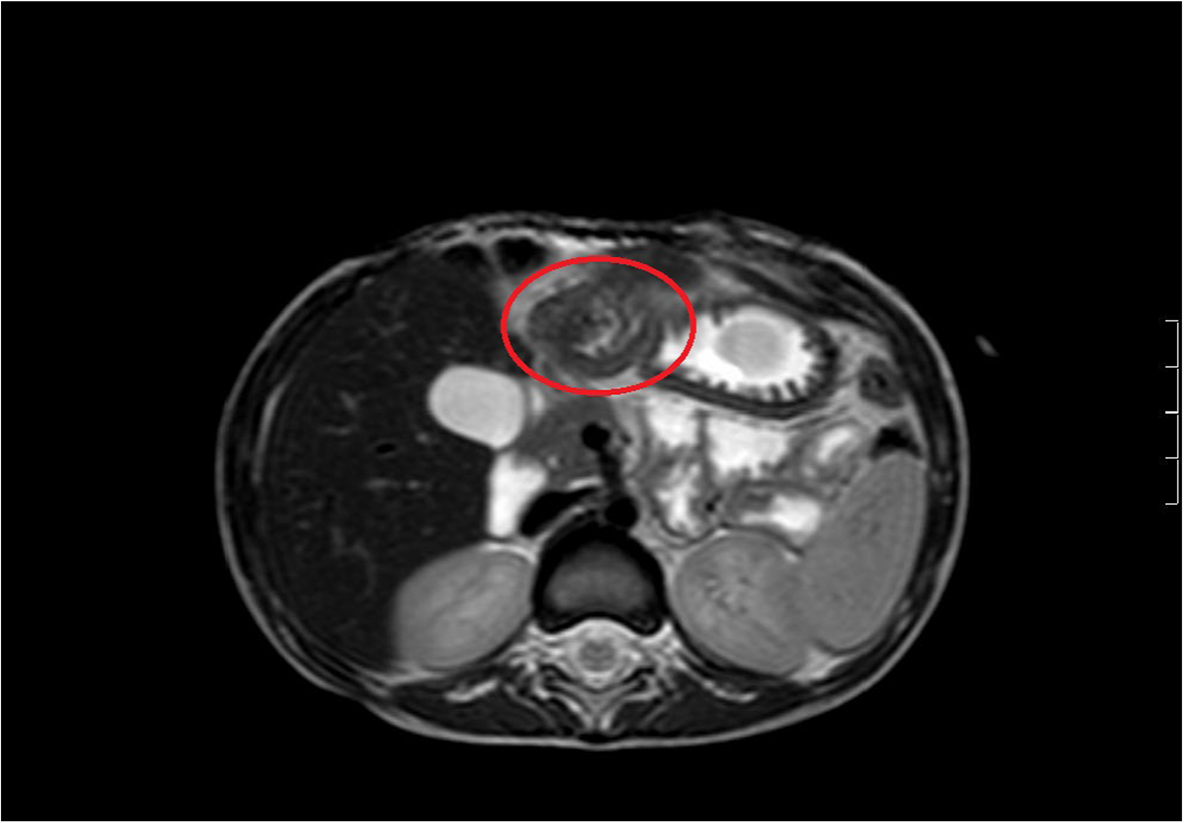
Magnetic resonance imaging of gastro-pyloric heterotopic pancreas (circle)

**Fig. 2:**
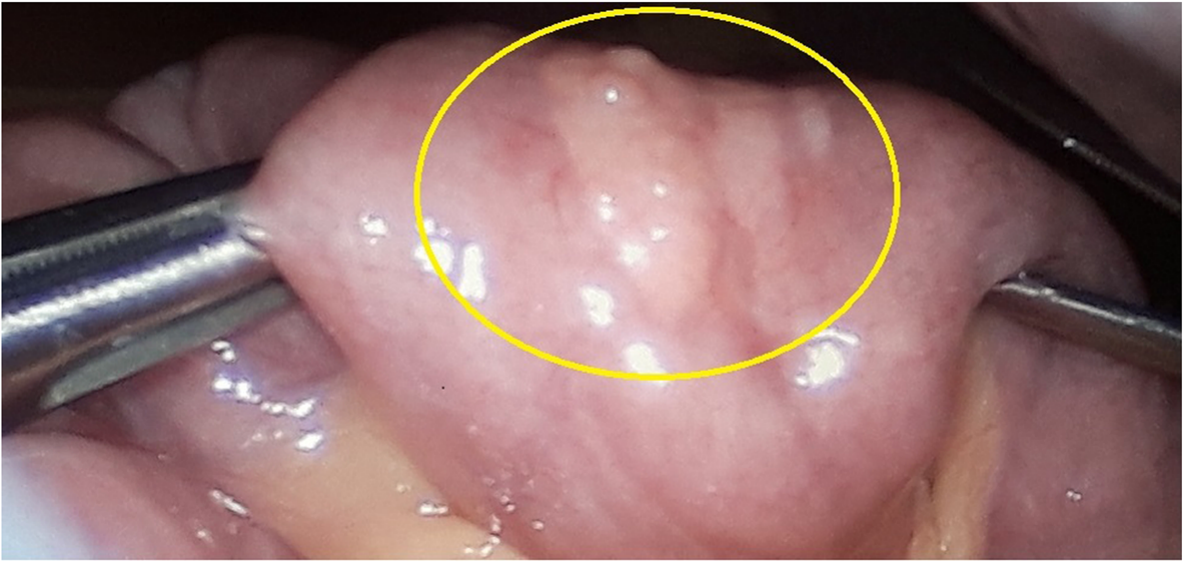
Intraoperative videolaparoscopic image of heterotopic pancreas on the jejunum (circle)

Seven patients were classified as “symptomatic” and seven patients were classified as “incidental”. Only two symptomatic patients had pre-operative magnetic resonance imaging suggestive of pathological tissue *(*Fig. 1*)*; one of them received laparotomy, the other underwent videolaparoscopy *(*Fig. 2*)*. In all other cases, diagnosis of abnormal tissue was made intraoperatively (6 laparotomies, 5 videolaparoscopies, 1 esophago-gastro-duodenoscopy) and confirmed by histology.

Patients’ age ranged from 2 months to 16 years. Four patients were younger than 1 year while ten patients were older; all the symptomatic patients were older than 1 year (70% of the patients older than 1 year). Mean age of symptomatic patients was 9 years and 5 months, while incidental patients’ mean age was 2 years and 9 months; symptomatic patients were significantly older than “incidental” (*p* = 0.02). Patients’ characteristics are summarized in Table 1.

**Table 1:** Patients’ characteristics

Patient	Age	Clinical presentation	Presenting complaint	Localization	Pre-op diagnosis	Surgery	Timing of surgery
1	2 months	Incidental	Midgut volvulus secondary to congenital malrotation	Small bowel	No	Open	Urgent
2	2 months	Incidental	Obstruction secondary to duodenal duplication	Duplication	No	Open	Urgent
3	2 months	Incidental	Recurrent vomiting secondary to congenital malrotation	Meckel’s diverticulum	No	Open	Elective
4	6 months	Incidental	Intussusception secondary to duplication	Duplication	No	Open	Urgent
5	1 year 9 m	Incidental	Intussusception secondary to Meckel’s diverticulum	Meckel’s diverticulum	No	Open	Urgent
6	2 years 4 m	Symptomatic	Melena and anemia	Small bowel	No	VLS	Urgent
7	3 years	Symptomatic	Recurrent vomiting secondary to gastric outlet obstruction	Gastro-pyloric	MRI	Open	Elective
8	4 years	Symptomatic	Intussusception secondary to small bowel mass	Small bowel	No	Open	Urgent
9	5 years	Incidental	Internal hernia secondary to Meckel’s diverticulum	Meckel’s diverticulum	No	VLS	Urgent
10	11 years	Incidental	Intussusception secondary to Meckel’s diverticulum	Meckel’s diverticulum	No	VLS	Urgent
11	12 years	Symptomatic	Recurrent abdominal pain	Small bowel	MRI	VLS	Elective
12	13 years	Symptomatic	Melena and anemia	Meckel’s diverticulum	No	VLS	Urgent
13	15 years	Symptomatic	Dyspepsia and weight loss	Gastro-pyloric	No	UGI endo	Elective
14	16 years	Symptomatic	Melena and recurrent abdominal pain	Meckel’s diverticulum	No	VLS	Elective

*MRI* Magnetic Resonance Imaging, *VLS* Videolaparoscopy, *UGI endo* upper gastrointestinal endoscopy

Five patients underwent elective procedures; four patients were found to have symptomatic heterotopic pancreas. In the same years, 2256 underwent elective laparotomy or videolaparoscopy for various indications; in this population, the incidence of heterotopic pancreas is 0.22%. Nine patients underwent urgent surgery for volvulus, intussusception, acute obstruction or gastrointestinal bleeding with acute anemization; in three patients, symptoms were caused by heterotopic pancreas. Over the same period, a total of 344 urgent surgical procedure were performed for the same indications; in this population, the incidence of heterotopic pancreas is 2.61%. The incidence of heterotopic pancreas in patients undergoing urgent procedures was significantly higher than in patients undergoing elective surgery (*p* < 0.0001).

All the patients underwent resection of the heterotopic pancreas. Two patients were classified as gastro-pyloric: both were symptomatic (100%) and are symptom-free at a follow-up of 1 and 3 years, respectively. Four patients were classified as small bowel and three of them were symptomatic (75%); one patient, who underwent laparotomy for neonatal midgut volvulus, experienced small bowel obstruction that required a second laparotomy, two patients are symptom-free and one patient was lost at follow-up. Six patients were classified as Meckel’s diverticulum and two were symptomatic (33%); one patient, who underwent laparotomy at 2 months, has occasional episodes of abdominal pain, while all the others are symptom-free. Two patients were classified as duplication and none was symptomatic (0%); the patient with duodenal duplication has asymptomatic persistently elevated pancreatic enzymes, while the other patient had an episode of severe constipation that was treated conservatively 1 month after the operation. Correlation between clinical presentation and localization was not statistically significant for any anatomical site; data are summarized in Table 2.

**Table 2:** Correlation between clinical presentation and localization

Clinical presentation	Gastro-pyloric	Small bowel	Meckel’s diverticulum	Duplication
Incidental	0	1	4	2
Symptomatic	2	3	2	0
% symptomatic	100	75	33	0
*p*-value	0.46	0.56	0.59	0.46

Eight patients (58%) were diagnosed with type I heterotopia: two patients had the heterotopic pancreas in the gastro-pyloric area, three patients in the small bowel, two patients in the Meckel’s diverticulum and one in the duodenal duplication. One patient (7%) was diagnosed with type II heterotopia in the small bowel. Two patients (14%) had heterotopic pancreas with diffuse necrotic and hemorrhagic alterations in the Meckel’s diverticulum; in both patients, heterotopic tissue was found incidentally during urgent surgery for mechanical complications of the Meckel’s diverticulum. Of interest, three patients (21%) presented with associated gastric and pancreatic heterotopia, two in the Meckel’s diverticulum and one in an intestinal duplication. Data are summarized in Table 3.

**Table 3:** Histology

Patient	Age	Clinical presentation	Localization	Histology
1	2 months	Incidental	Small bowel	Type I
2	2 months	Incidental	Duplication	Type I
3	2 months	Incidental	Meckel’s diverticulum	Type I
4	6 months	Incidental	Duplication	Gastric-pancreatic heterotopia
5	1 year 9 months	Incidental	Meckel’s diverticulum	Necrotic-Hemorragic
6	2 year 4 months	Symptomatic	Small bowel	Type I
7	3 years	Symptomatic	Gastro-pyloric	Type I
8	4 years	Symptomatic	Small bowel	Type II
9	5 years	Incidental	Meckel’s diverticulum	Necrotic-Hemorragic
10	11 years	Incidental	Meckel’s diverticulum	Gastric-pancreatic heterotopia
11	12 years	Symptomatic	Small bowel	Type I
12	13 years	Symptomatic	Meckel’s diverticulum	Gastric-pancreatic heterotopia
13	15 years	Symptomatic	Gastro-pyloric	Type I
14	16 years	Symptomatic	Meckel’s diverticulum	Type I

There was no statistically significant correlation between histology and localization of the heterotopic tissue.

## Discussion

The pathogenesis of heterotopic pancreas in the gastrointestinal tract is still debated; one of the two main theories speculates that embryonic cells could be transplanted to adjacent structures during axial rotation of the intestine [13]. According to a second theory, heterotopic tissue may arise from in situ differentiation of multipotent cells [14, 15].

Heterotopic pancreas is usually discovered in the submucosa of the upper gastrointestinal tract, including the stomach, duodenum, and in smaller proportions in the jejunum, ileum and Meckel’s diverticulum; it is rarely found in the esophagus, liver, gallbladder, biliary tree, spleen, omentum, lungs, mediastinum, fallopian tubes or even umbilicus [4, 16, 17]. In our case series only 3 patients out of 14 (21,4%) had heterotopic tissue localized proximal to the ligament of Treitz; this in contrast with the general literature [4, 16, 17] but consistent with a previous pediatric study, that reports most cases of heterotopic pancreas in the Meckel’s diverticulum and in the small bowel distal to the duodeno-jejunal junction [8]. Moreover, some authors report a higher proportion of heterotopic pancreas in the ileum in recent years compared to their previous case series; these authors suggested that there might be an increase in the incidence of heterotopic pancreas in the ileum [7]. We speculate that a different explanation could be the development of new techniques, such as videolaparoscopy and capsule endoscopy, which allow minimally invasive exploration of the small bowel, thus improving the ability to diagnose heterotopic pancreas in the distal jejunum and the ileum [18].

When located in the stomach or in the duodenum, the presence of heterotopic pancreas can be suspected based on typical radiographic findings on upper gastrointestinal series. Heterotopic pancreas can be identified as a mass with broad base and smooth surface characteristic of extra-mucosal intramural tumours. Umbilication, manifested by pooling of barium, is thought to represent the ductal remnant. Visualization of a barium-filled pit at the centre of the lesion permits the specific diagnosis of heterotopic pancreatic tissue [19]. On magnetic resonance imaging, heterotopic pancreas appears as hyperintense or isointense lesions compared with the normal pancreas on unenhanced T1-weighted images and isointense or hypointense lesions compared with the pancreas on T2-weighted images. On dynamic MRI, heterotopic pancreas appears as isointense lesions compared with the normal pancreas on arterial phase images [20]. Radiologic findings in case of heterotopic pancreas distal to the duodeno-jejunal junction are non specific [19].

Foci of heterotopic pancreas are usually diagnosed incidentally [4, 5]. Heterotopic tissue may cause symptoms related to mechanical complications, such as gastric outlet obstruction [9] or intussusception [21, 22]; in other circumstances, heterotopic pancreatic tissue may cause bleeding from the surrounding intestinal mucosa [23]. Occasionally, metaplastic changes may occur in heterotopic tissue, leading to the development of malignancy in adulthood [5, 24, 25]; even though exceedingly rare, malignancies arising from heterotopic pancreas have been described also in children [26–28]. In our series, 7 out of 14 patients (50%) had symptoms related to the presence of heterotopic pancreas; presenting complaint were recurrent vomiting, dyspepsia, intussusception, bleeding and recurrent abdominal pain.

All the symptomatic patients were older than 1-year; to our knowledge, only one article reported symptomatic heterotopic pancreas in two children younger than 1-year (4 months and 6 months, respectively) [9], while other authors reported incidental findings of heterotopic pancreas in neonates undergoing abdominal surgery for other indications [8]. The rarity of neonates and infants who present symptoms related to heterotopic pancreas could be related to the rarity of the condition itself or to the possibility that foci of heterotopic pancreas increase in size with the growth of their hosts, thus explaining cases of late onset of symptoms [3, 29]; our data show that symptomatic patients are significantly older than incidental, thus supporting the latter theory [3, 29].

Patients undergoing urgent abdominal surgery were found to have foci of heterotopic pancreas more frequently than patients undergoing elective surgery (2.61 vs 0.22, *p* = 0.02); our results could be related to the fact that, during the study period, 180 out of 344 (52,3%) urgent surgical procedures were motivated by intussusception or gastrointestinal bleeding with acute anemization, that are well described clinical manifestations of heterotopic pancreas^21–23^.

In the literature, gastro-pyloric heterotopic pancreas tends to cause symptoms more frequently than other localization [4, 7, 9]; although all the gastro-pyloric heterotopies in our series were symptomatic, statistical analysis of our data did not show any significant difference, probably due to the small sample size.

In the present series, three patients were diagnosed with associated gastric and pancreatic heterotopia, two in the Meckel’s diverticulum and one in a small bowel duplication. The presence of combined heterotopic tissue has been reported both in Meckel’s diverticulum [30] and in intestinal duplication [31]; the clinical implications of such association are poorly understood and deserve further investigation.

Due to the rarity of the condition, optimal management of heterotopic is still debated. All authors agree that symptomatic heterotopic tissue should be resected^3–9^; in asymptomatic patients, some authors suggest surgical excision to avoid the risk of late complications [8], whilst others advocate close monitoring of asymptomatic patients whose lesions are found incidentally, once a histopathological diagnosis has been obtained [3]. In our experience, the intra-operative finding of heterotopic tissue is difficult to interpret based on macroscopic appearance alone; considering the risk of severe complications and the need of a clear histopathological diagnosis, we advise the resection of foci of heterotopic pancreas whenever they are found.

The present study is retrospective in nature and comprises a small number of patients; therefore, limited evidence can be obtained and strong recommendation for management cannot be formulated. On the other hand, the rarity of heterotopic pancreas limits the possibility for prospective studies.

## Conclusion

The presence of heterotopic pancreas should be considered in pediatric patients with gastrointestinal symptoms of unclear origin, especially in the setting of acute presentation. The resection of heterotopic tissue is advisable in order to obtain a histological diagnosis and to avoid future complications.

## Data Availability

The dataset supporting the conclusions of this article is included within the article (and its additional files).

## Abbreviations

MRI: Magnetic Resonance Imaging
UGI endo: Upper gastrointestinal endoscopy
VLS: Videolaparoscopy
